# New-onset dermatomyositis following COVID-19: A case report

**DOI:** 10.3389/fimmu.2022.1002329

**Published:** 2022-10-24

**Authors:** Hiroshi Shimizu, Haruki Matsumoto, Tomomi Sasajima, Tomohiro Suzuki, Yoshinori Okubo, Yuya Fujita, Jumpei Temmoku, Shuhei Yoshida, Tomoyuki Asano, Hiromasa Ohira, Yutaka Ejiri, Kiyoshi Migita

**Affiliations:** ^1^ Department of Gastroenterology, Fukushima Rosai Hospital, Iwaki, Japan; ^2^ Department of Rheumatology, Fukushima Medical University School of Medicine, Fukushima, Japan; ^3^ Department of Rheumatology, Fukushima Rosai Hospital, Iwaki, Japan; ^4^ Department of Gastroenterology, Fukushima Medical University School of Medicine, Fukushima, Japan

**Keywords:** anti-synthetase antibodies, anti-aminoacyl tRNA synthetase (ARS) antibodies, autoimmune diseases, dermatomyositis, COVID-19

## Abstract

Coronavirus disease 2019 (COVID-19) is an infectious disease caused by severe acute respiratory syndrome coronavirus 2 (SARS-CoV-2). Most of the infected individuals have recovered without complications, but a few patients develop multiple organ involvements. Previous reports suggest an association between COVID-19 and various inflammatory myopathies, in addition to autoimmune diseases. COVID-19 has been known to exacerbate preexisting autoimmune diseases and trigger various autoantibodies and autoimmune disease occurrence. Here we report a case of complicated COVID-19 with anti-synthetase autoantibodies (ASSs) presenting with skin rash, muscle weakness, and interstitial lung disease (ILD) and subsequently diagnosed with dermatomyositis (DM). A 47-year-old Japanese male patient without any previous history of illness, including autoimmune diseases, presented with a high fever, sore throat, and cough. Oropharyngeal swab for SARS-Cov-2 polymerase chain reaction tested positive. He was isolated at home and did not require hospitalization. However, his respiratory symptoms continued, and he was treated with prednisolone (20 mg/day) for 14 days due to the newly developing interstitial shadows over the lower lobes of both lungs. These pulmonary manifestations remitted within a week. He presented with face edema and myalgia 4 weeks later when he was off corticosteroids. Subsequently, he presented with face erythema, V-neck skin rash, low-grade fever, and exertional dyspnea. High-resolution computed tomography of the chest showed ILD. Biochemical analysis revealed creatine kinase and aldolase elevations, in addition to transaminases. Anti-aminoacyl tRNA synthetase (ARS) was detected using an enzyme-linked immunosorbent assay (170.9 U/mL) (MESACUP™ (Medical & Biological Laboratories, Japan), and the tRNA component was identified as anti-PL-7 and anti-Ro-52 antibodies using an immunoblot assay [EUROLINE Myositis Antigens Profile 3 (IgG), Euroimmun, Lübeck,Germany]. The patient was diagnosed with DM, especially anti- synthase antibody syndrome based on the presence of myositis-specific antibodies, clinical features, and pathological findings. The present case suggests that COVID-19 may have contributed to the production of anti-synthetase antibodies (ASAs) and the development of *de novo* DM. Our case highlights the importance of the assessment of patients who present with inflammatory myopathy post-COVID-19 and appropriate diagnostic work-up, including ASAs, against the clinical features that mimic DM after post-COVID-19.

## Introduction

Coronavirus disease 2019 (COVID-19), which is caused by the severe acute respiratory syndrome coronavirus 2 (SARS-CoV-2), is a life-threatening respiratory illness (1). COVID-19 is a heterogeneous disease ranging from asymptomatic course to multi-organ failure during the inflammatory processes (2). In addition, COVID-19 shares clinical similarities with autoimmune diseases, and some patients have been reported to develop these autoimmune diseases (3). Moreover, clinical similarities had been suggested between COVID-19 and anti-melanoma differentiation-associated protein 5 (MDA5)-positive dermatomyositis (DM) (4). Robust activations of immune systems participate in the pathophysiological mechanisms of both disease conditions (5). The main pathophysiological mechanisms for severe inflammation and organ damage seen in patients with COVID-19 are thought to be immune activation and proinflammatory cytokine induction (6). Indeed, elevated serum levels of proinflammatory cytokines, including interleukin (IL)-1β, IL-16, IL-8, and IL-18, were demonstrated in patients with COVID-19 (7). Other clinical features of COVID-19 infection were reported as these viral infections are postulated to induce autoimmunity (8). Various autoantibodies have been detected in the serum of patients with COVID-19, including anti-nuclear antibodies (ANA) and anti-phospholipid antibodies (9). DM is an autoimmune disease in which the skin and muscles are the targets for immune-mediated destruction (10). This inflammatory myopathy can be complicated by vasculopathy and interstitial lung disease (ILD) (11). Autoantibodies, as a hallmark of autoimmune diseases, can also be detected transiently in patients with COVID-19 (12). Antibodies that recognize different amino tRNA synthase serve as the serological hallmark of the anti- synthase antibody syndrome (ASS) that consists of myositis, ILD, mechanic’s hands and fever (13). A higher prevalence and increased severity of ILD were found in patients with ASS than those with DM and polymyositis (14). Here, we report a Japanese patient who presented with DM with skin rash, proximal limb weakness, and seropositivity of ASSs after COVID-19 infection. We focused on the new-onset anti- synthase antibody (ASA)-related DM and COVID-19 with a recent literature review.

## Case description

**Figure 1:**
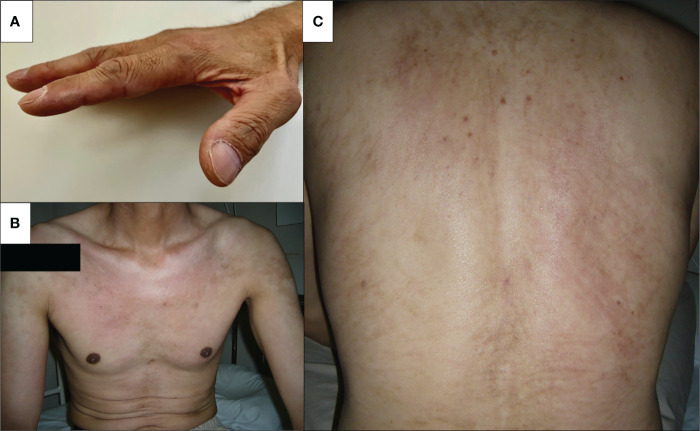
Skin findings on admission. Physical examination revealed skin manifestation of dermatomyositis. **(A)** Mechanic’s hand, **(B)** V-neck sign on the chest, and **(C)** whiplash-like erythema on the back.

**Figure 2:**
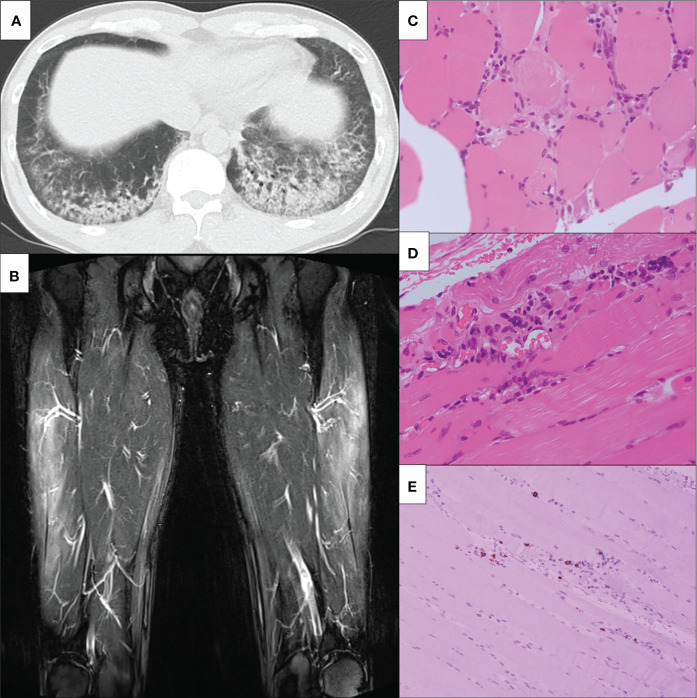
Clinical imagings. **(A)** Chest non-contrast CT findings. Chest non-contrast CT revealed bilateral ground-glass opacity. **(B)** MRI findings of bilateral lower limbs. MRI shows high signals on STIR in the bilateral vastus lateralis, suggesting muscular inflammation. MRI, magnetic resonance imaging; STIR, short T1 inversion recovery. **(C-E)** The slide showed **(C)** inflammatory cell infiltration around the myofiber bundles, **(D)** perivascular inflammatory cell infiltration, and **(E)** CD8-positive lymphocytes infiltration in atrophy of the myofibers. CT, computed tomography; HE, hematoxylin-eosin; MRI, magnetic resonance imagin; STIR, short T1 inversion recovery; CD, cluster of differentiation.

A 47-year-old Japanese male patient presented with persistent low-grade fever, malaise, and cough after the once disappearance of the COVID-19-related symptoms 28 days from SARS-CoV-2 RNA detection by polymerase chain reaction. The patient had received the second dose of mRNA-1273 SARS-CoV-2 vaccination (Moderna), 5 months before the detection of SARS-CoV-2 RNA without any acute side effects. He received only symptomatic treatment, and these manifestations lasted for 10 days. Three weeks later from the SARS-CoV-2 RNA, the erythematous skin macules appeared on the patient’s eyelid, which became more intense and extended to the anterior chest. He was referred to our Respiratory Medicine Department due to the sustained respiratory sympotoms. On first visit in our hosipital, he presented eyelid edema and skin rash on both upper extremities, chest, and back (**Figures 1A–C**). Chest computed tomography (CT) was performed since he had a history of nontuberculous mycobacterial disease. A CT scan revealed interstitial pneumonia (**Figure 2A**), and he was treated with antimicrobial agents. Concurrently, he visited our outpatient dermatology clinic for eyelid edema treatment and was transiently treated with prednisolone (PSL) at 20 mg for 14 days. Respiratory symptoms and eyelid edema improved with oral PSL administration; thereafter, PSL was discontinued. However, he presented with more severe dyspnea, cough, dysarthria, dysphagia, odynophagia, and severe generalized weakness with inability to ambulate, elevated transaminases, and relapsing interstitial pneumonia 3 weeks later. He was started on PSL at 10 mg and admitted to the hospital for further examinations for elevated transaminases, high immunoglobulin (Ig)G levels, and interstitial pneumonia.

Physical examination was significant for tachycardia to 110 beats per minute and oxygen saturation of 94% on room air. On examination, eyelid edema had resolved; however, mechanic’s hands, V-neck skin rash, and whiplash-like erythema on the back were noted.

**Figure 3:**
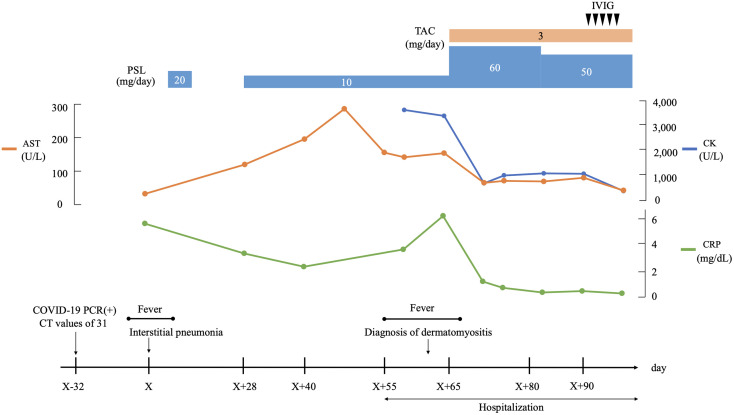
Clinical course. AST, aspartate aminotransferase; CK, creaine kinase; COVID-19, coronavirus disease 2019; CRP, C-reactive protein; CT, cycle threshold; IVIG, intravenous immunoglobulin; PCR, polymerase chain reaction; PSL, prednisolone; TAC, tacrolimus.

His proximal muscle power was 4/5 bilateral on the upper and lower limbs. Laboratory data showed positive ANA (speckled and cytoplasmic patterns at a serum dilution of 1:1280 and nucleolar pattenr at a serum dilution of 1:320 by indirect immunofluorescence) and were associated with significantly increased muscle enzymes [creatinine phosphokinase of 3,380 U/L (62-287 U/L)]. He was positive with high titers for anti-Ro/SSA Ab(>240 U/mL; normal range: <6.7) and anti-La/SSB (>240 U/mL; normal range: <6.7); however, negative results for the Saxon and Schirmer tests were observed. Anti-ARS antibodies were positive with high titers [170.9 U/mL (<24.9 U/mL)] according to the findings of an enzyme-linked immunosorbent assay (MESACUP™ (Medical & Biological Laboratories, Japan). We further investigated the autoantigen of anti-ARS antibody by immunoblot assay [EUROLINE Myositis Antigens Profile 3 (IgG), Euroimmun, Lübeck,Germany], which thus revealed positivity (3+) for anti-PL-7 antibody. In HLA-DRB1 gene analysis, he had DR4 (DRB1*0405)) and HLA-DR15 (DRB1*15:01). (**Supplemental Table 1**). Magnetic resonance imaging (MRI) presented diffuse edema and inflammatory changes in the bilateral thigh muscles (**Figure 2B**). On pathologic examination, there was moderate size variation of the myofibers, and CD8-positive lymphocytes infiltration in atrophy of the myofibers, mainly at the periphery of the fascicles (**Figures 2C–E**). He was diagnosed with ASS according to criteria proposed by Lega JC, et al. (15). Treatment was started with 60 mg of PSL and 3 mg of tacrolimus per day. Fatigue, fever, and myalgia quickly improved post-treatment, whereas serum creatine kinase (CK) levels were not completely normalized during the PSL tapering phase; thus, a high dose of intravenous immunoglobulin (IVIG) was added. The patient’s muscle strength improved with muscle enzyme level normalization after these combined treatments. Blood analysis revealed sustaining normal levels of muscle enzymes, and the patient remains in close medical observation at our out-patient clinic. The clinical course is summarized in **Figure 3**.

## Discussion

ASS is an inflammatory myopathy caused by ASAs. Its clinical presentations are characterized by ILD, myositis, polyarthritis, fever, and “mechanic’s hands” (16). Immuno-genetic study of HLA-DRB1 associations performed in cohort of Caucasian patients with ASS. In this study, HLA-DRB1*03:01 allele was identified as predisposition markers of ASS and HLA-DRB1*07:01 had a protective effect in the susceptibility to ASS. Our case showed both HLA-DR15 (DRB1*15:01) and DR4 (DRB1*0405), which may not affect the susceptibility for ASS in the present case (17). COVID-19 infection may present with a multitude of pulmonary findings, including diffuse ground-glass opacities (18). Similarly, the pulmonary manifestations of an ASS may present with patchy ground-glass opacities, similar to the interstitial shadows commonly seen in COVID-19-related pneumonia (19). The inflammatory process caused by SARS-CoV-2 infection involves cytokine storm and macrophage activation (20). Therefore, COVID-19 shared clinical features with rheumatic diseases characterized by elevated inflammatory cytokine levels. Here, we report a patient with COVID-19 who was later associated with DM that involves the proximal limb muscles and typical cutaneous manifestations, including V-neck skin ra. Our case describes a newly diagnosed anti-PL-7-positive DM complicated by ILD with a recent COVID-19 diagnosis. Some cases are reported to develop autoimmune diseases after COVID-19 (21). The overall incidence of COVID-19 associated with DM remains rare. COVID-19 had resulted in flares of preexisting rheumatic diseases (22); however, this is unlikely since no clinical symptoms were suggesting rheumatic manifestation before the onset of COVID-19 in the present case. Whether COVID-19-induced viral myositis mimics DM-like clinical manifestations or DM itself can be argued. The time interval between the onset of erythematous skin rash and the COVID-19 RNA detection is 3 weeks; thus, it can be presumed to be new-onset DM and not COVID-19-related myositis. In addition, the seroconversion of ASSs supports this idea; however, whether autoantibody positivity is persistent or transient should be evaluated. The newly appearing radiographic finding of nonspecific interstitial pneumonia (NSIP)-like bilateral ground-glass opacities also supports ASA-positive DM-associated ILD. Proinflammatory clinical manifestation seen as COVID-19 complications mimics the symptoms of DM (23). However, we should discriminate between real DM and post-viral myositis following SARS-CoV-2 infection.

SARS-CoV-2 may cause postinfectious myositis, which may range from direct virus-induced myositis to virus-triggered autoimmunity-related myositis (24). Whether COVID-19 contributes to the occurrence of typical DM that carries the myositis-specific autoantibodies remained unclear. As postulated, autoimmune mechanisms can be developed as a consequence of the molecular autoantigen transformation or modifications due to the influence of the SARS-CoV-2 infection (25). However, a limited number of case reports demonstrated the occurrence of myositis with specific autoantibodies as a consequence of the clinical course of COVID-19 (23). Idiopathic inflammatory myopathy is one of the potential autoimmune diseases that could be triggered by COVID-19 (23). These patients have both DM and COVID-19 presented with various cutaneous manifestations, elevated CK, and partly associated with seropositivity for myositis-specific antibodies (MSAs).

Viral infections may serve as a trigger although the association between COVID-19 and DM development remains unclear. A recent epidemiological survey suggests that the increasing number of patients with autoimmune diseases coincides with the COVID-19 pandemic (21). SARS-CoV-2 has been speculated to break the self-tolerance and trigger autoimmune responses through inflammatory cytokine induction (21). Dysregulation of neutrophil extracellular trap (NET) formation has been shown to mediate disease pathology in multiple viral infections (26), including SARS-CoV-2. Indeed, dysregulation of NET formation has been demonstrated in SARS-CoV-2 infection (27). Therefore, the complexity of COVID-19 and of NET formation may relate to the autoantibodies production through following an adaptive immune response. The clinical features of severe COVID-19 are postulated to be similar to those of anti-MDA-5-positive DM (28), which may suggest the immune-mediated mechanisms for these disorders. Type 1 interferon (IFN) signature has been implicated in the pathogenesis of autoimmune diseases, including rheumatic diseases (29). COVID-19 pathogenesis may include the induction of a hyper-inflammatory state with elevated inflammatory cytokines, including type 1 IFN, which leads to autoantibody induction (30). Another mechanism includes the molecular mimicry between viral antigen and damaged muscle antigen leading to the adaptive immune system producing autoantibodies (31). Therefore, determining the coexistent COVID-19 and DM with definitely diagnosed patients with DM with myositis-specific autoantibodies and typical histological manifestations is important to elucidate the immunopathology for COVID-19-related DM.

Myositis-specific autoantibodies are an important clue for DM diagnosis (32). Recent studies identified three immunogenic linear epitopes with coronavirus 2 (SARS-CoV-2) proteins in anti-TIF1-γ DM, which suggest the possibility of overlapping COVID-19 and DM (33). Newly diagnosed anti-MDA-5-positive DM following the recent COVID-19 diagnosis was reported (28). Furthermore, anti-MDA-5-Ab is frequently detected in patients with COVID-19, and its titers correlate with severe disease and poor COVID-19 outcomes (34). However, the coincidence between COVID-19 and the ASS is rarely reported. Blake T et al. reported a case of a patient with COVID-19-like pneumonia who was positive for anti-PL-7 antibody, but without virological evidence of COVID-19 (35). **Table 1** shows cases with virologically proven COVID-19 infection complicated with dermatomyositis with MSAs excluding Anti-MDA5-Ab, suggesting a fair prognosis for these coexisting patients (32–36). In contrast to the other MSAs, such as an anti-MDA5 antibody, the associations between the other MSAs-positive DM and COVID-19 were limited, which may indicate the necessity to further evaluate MSAs in patients with COVID-19-related myopathy to elucidate their true relationships.

**Table 1:** Complicated cases of COVID-19 and myositis with MSAs excluding anti-MDA5 Ab.

Authors	Year	Sex	Age of onset	Antibody of PM/DM	ILD	Symptoms of myositis	Skin rash	Outcomes
Zhang et al. (36)	2020	F	58	Anti-SAE, Ku Ab	NA	Bilateral ptosis, facial weakness, hypernasal dysarthria, and proximal limb weakness	NA	Recovered
Sacchi et al. (37)	2020	F	77	Anti-Ku, Mi-2 Ab	YES	Aprexia	NA	Recovered
Borges et al. (38)	2021	F	36	Anti-Mi-2 Ab	NA	Proximal limb weakness	Gottron’s papules	Recovered
Okada et al. (39)	2021	F	64	Anti-NXP2 Ab	No	Neck and proximal limb weakness	Itchy erythematous lesions on forehead, bilateral ears, scalp, and neck, lacking typical heliotrope rash or Gottron’s sign Periungual telangiectasias	Recovered
Faria et al. (40)	2022	F	59	Anti-Mi-2, SAE Ab	YES	Limb weakness	Heliotrope rash, Gottron’s sign with ulcerations, and cuticular hypertrophy	Recovered

COVID-19, coronavirus disease 2019; MSA, myositis specific antibodies; PM, polymyositis; DM, dermatomyositis; ILD, interstitial lung disease; F, female; Ab, antibody; NA, not applicable.

COVID-19 is a novel pandemic that has significant concern on the occurrence of various inflammatory disorders and subsequent organ damage. A possible linkage was found between COVID-19 and autoimmune diseases through the immune-mediated inflammatory pathways. The present case report suggests that COVID-19 infection may trigger the SSA-related DM. More data are needed to elucidate the relationship between COVID-19 and the risk of the induction of MSAs and the occurrence of DM, and these patients possess a particular genetic susceptibility.

In conclusion, SARS-CoV-2 can induce myopathy in certain high risk hosts, which mimics the symptoms of DM. We reported a case of PL-7-positive DM in a patient with COVID-19, who responded to steroid plus immunosuppressive treatments. The link between COVID-19 and the development of ASS-related DM needs further investigation, and clinicians should remain vigilant about potential muscle involvement post-COVID-19. The natural history and prognosis or these patients compared to their *de novo* DM counterparts remain unclear.

## Data availability statement

The original contributions presented in the study are included in the article/**Supplementary Material**. Further inquiries can be directed to the corresponding author.

## Ethics statement

Written informed consent was obtained from the individual(s) for the publication of any potentially identifiable images or data included in this article.

## Author contributions

HS, HM, YF, JT, SY, TA, HO and KM were involved with the conception of the work. HS, TSa, TSu, YO, and YE contributed to the treatment and collection of data. HS, HM and KM wrote the first draft of the manuscript. All authors contributed to the article and approved the submitted version.

## Acknowledgments

We would like to thank Enago (www.enago.jp) for English language review.

## Conflict of interest

The authors declare that the research was conducted in the absence of any commercial or financial relationships that could be construed as a potential conflict of interest.

## Publisher’s note

All claims expressed in this article are solely those of the authors and do not necessarily represent those of their affiliated organizations, or those of the publisher, the editors and the reviewers. Any product that may be evaluated in this article, or claim that may be made by its manufacturer, is not guaranteed or endorsed by the publisher.
